# Duck Plague Virus Negatively Regulates IFN Signaling to Promote Virus Proliferation *via* JNK Signaling Pathway

**DOI:** 10.3389/fimmu.2022.935454

**Published:** 2022-06-28

**Authors:** Liping Wu, Bin Tian, Mingshu Wang, Anchun Cheng, Renyong Jia, Dekang Zhu, Mafeng Liu, Qiao Yang, Ying Wu, Juan Huang, XinXin Zhao, Shun Chen, Shaqiu Zhang, Xumin Ou, Sai Mao, Qun Gao, Di Sun, Yanling Yu, Ling Zhang, LeiCHang Pan

**Affiliations:** ^1^Institute of Preventive Veterinary Medicine, Sichuan Agricultural University, Chengdu City, China; ^2^Key Laboratory of Animal Disease and Human Health of Sichuan Province, Sichuan Agricultural University, Chengdu City, China; ^3^Avian Disease Research Center, College of Veterinary Medicine, Sichuan Agricultural University, Chengdu City, China

**Keywords:** duck plague virus, duck monocytes/macrophages, RNA-seq, signal transduction pathway, JNK signaling pathway

## Abstract

Duck plague virus (DPV), a member of the *alphaherpesvirus* subfamily, can cause severe damage and immunosuppression in ducks and geese in China. Since lacking an available cell model, the antiviral signal transduction pathways induction and regulation mechanisms related to DPV infection in duck cells are still enigmatic. Our previous study developed a monocyte/macrophages cell model, which has been applied to study innate immunity with DPV. In the present study, we compared and analyzed transcriptome associated with the DPV infection of CHv (virulent strain) and CHa (avirulent strain) at 48hpi based on the duck monocyte/macrophages cell model and RNA-seq technology. Differentially expressed genes (DEGs) analysis showed 2,909 and 2,438 genes altered in CHv and CHa infected cells compared with control cells. Gene Ontology (GO) and Kyoto Encyclopedia of Genes and Genomes (KEGG) pathway enrichment analysis showed that the DEGs were mainly involved in biological processes such as metabolic pathways, viral infectious diseases, immune system, and signal transduction. The CHv and CHa virus differentially regulated MAPK, NF-κB, and IFN signaling pathways based on transcriptome sequencing data and RT-qPCR results. The JNK inhibitor SP600125 enhanced the IFN signaling, but potentially reduced the VSV and DPV titers in the cell culture supernatant, indicating that JNK negatively regulates the IFN pathway and the inflammatory pathway to promote virus proliferation. The research results may provide promising information to understand the pathogenesis of DPV and provide a novel mechanism by which DPV modulates antiviral signaling and facilitate virus proliferation through hijacking the JNK pathway, which provides a new means for the prevention and control of DPV infection.

## Introduction

Duck plague (also known as duck viral enteritis) was initially identified in 1957 in China by Yinxian Huang and is now prevalent in many duck-raising places worldwide (1). Duck plague is an acute, contagious, septic infectious disease among waterfowl (ducks, geese, swans of all ages and species) with high morbidity and mortality, and is a major hazard to the waterfowl industry (2). The DP causative agent, duck plague virus (DPV) or duck enteritis virus (DEV), is a member of the subfamily *Alphaherpesviruses*, whose genome consists of a linear double-stranded DNA, including a unique long region (UL), a unique short region (US), terminal short repeats (TR) and internal short repeats (IR), forming the genomic structure of UL-IRS-US-TRS (3). DPV Chinese virulent (CH virulent, CHv) strain, isolated from dead infected ducks in China, is highly pathogenic and could cause massive spotting hemorrhages in parenchymal organs, lymphoid and digestive tract, resulting in massive mortality of ducks (4–6). Immunization with DPV attenuated strain (CHa) is one of the most effective measures to control DP (7). Studies on DPV have been carried out for many years. However, the signaling pathways and antiviral mechanisms related to the infection of host cells by CHv strain and CHa strain are still unclear.

Cell signal transduction pathways regulate cell growth, cell survival, apoptosis, and immune responses. Mitogen-activated protein kinase (MAPK) cascades are crucial intracellular signaling pathways and transmit signals from the cell membrane to the nucleus, which is activated by a series of extracellular and intracellular stimuli, including growth factors, virus infection, bacterial complexes, cytokines, and various cellular stressors (8–10). MAPKs are involved in the cell survival stage, regulating intracellular metabolism, cell differentiation, gene expression, and integral activities in diverse cellular processes (11, 12). MAPK kinase kinases (MKKKs) activate MAPK kinases (MKKs) which in turn activate and phosphorylate MAPKs (13). Three major groups in the MAPKs cascades, such as p38, extracellular signal-regulated protein kinases (ERK), and c-Jun N-terminal kinases (JNK, also called stress-activated protein kinase/SAPK) (14).

Viruses can resort to modulating and hijacking the cellular signaling transduction pathways for efficient replication (15). For instance, HSV-1 infection stimulated PI3K/AKT and ERK MAPK signaling pathways that in turn contributed to KSHV reactivation (16); ERK activation mediated by KSHV virion enhances viral gene expression (17); HCV infection triggers TAB1-dependent p38 activation, which in turn phosphorylates the HCV core protein to promote HCV replication (18); FMDV infection induces the activation of MAPK signaling cascades, and inhibition of MAPK signaling pathway by MAPK pathway-specific inhibitor U1026 significantly impaired FMDV replication (19). However, little is known about the role of the MAPK signal pathway in DPV pathogenesis in duck cells.

In the present study, we applied the RNA-Seq method to analyze and compare the gene expression patterns in duck monocytes/macrophages infected by CHv strain and CHa strain at the late infection phase. The DEGs were mainly involved in biological processes such as metabolic pathways, viral infectious diseases, immune system, and signal transduction. We found that the MAPK inflammatory signaling pathway was significantly activated by CHv strain and CHa strain. Unexpectedly, the JNK inhibitor SP600125 significantly enhanced the IFN signaling under VSV and DPV infection, but potentially inhibited the replication of VSV and DPV, indicating that JNK negatively regulates the IFN pathway and the inflammatory pathway to promote virus proliferation. Our data suggested that JNK signaling is a promising target for DPV control. These results may provide information that will increase our understanding of DPV pathogenesis and the mechanisms underlying virus-host interactions.

## Materials and Methods

### Ethics Statement

All animal experiments were performed following approved guidelines. One-month-old Peking ducklings were conducted from a DPV-free farm, where vaccination against DPV was not implemented. All the ducks were housed in the animal facility at Sichuan Agricultural University in Chengdu, China. This study was approved by the Experimental Procedures and Animal Welfare Committee of Sichuan Agricultural University (approval permit number SYXK 2019-187).

### Cells, Virus Stains, and Reagents

Duck embryo fibroblast (DEF) cells were obtained from 9-day-old duck embryos. DEF and baby hamster kidney (BHK21) cells were cultured in Dulbecco’s Modified Eagle Medium (DMEM) (Gibco Life Technologies, Shanghai, China) supplemented with 10% fetal bovine serum(FBS; Gibco, USA)and 1% penicillin-streptomycin. Duck monocytes/macrophages were isolated, cultured, and identified as described earlier (20). Briefly, duck PBMCs were isolated using the Duck Leukocyte Isolation Kit (TBD science, Tianjin, China), plated on cell culture plates for 2 h, washed three times with PBS to remove non-adherent cells, and cells were digested with trypsin for cell counting. Duck monocyte-derived macrophages were differentiated from adherent monocytes in RPMI 1640 medium supplemented with l -glutamine (2 mM), sodium pyruvate (1 mM), 10% heat-inactivated fetal bovine serum, 1% penicillin-streptomycin and 50 ng/ml human M-CSF (Novo protein, Shanghai, China). The medium was changed every two days and the duck macrophages formed a monolayer on day 7. All cells were incubated at 37°C in a 5% CO2 humidified incubator.

Highly virulence DPV CHv strain, attenuated modified vaccine DPV CHa strain and the recombinant VSV strain harboring GFP (VSV-GFP) were obtained from the Key Laboratory of Animal Disease and Human Health of Sichuan Province. DPV strains were propagated and titrated on DEFs. VSV-GFP was propagated and titrated on BHK21.

SP600125 (c-Jun NH2-terminal kinase [JNK] inhibitor, purity 99.55%), LY3214996(extracellular signal-regulated kinase [ERK] inhibitor, purity 99.85%), TAK-715 (p38 inhibitor, purity 99.89%), BAY-11-7802 (NF-kB inhibitor, purity 99.98%), and Ruxolitinib (interferon receptor inhibitor) were purchased from MedChemExpress (Monmouth Junction, NJ). All inhibitors were diluted in dimethyl sulfoxide (DMSO).

### RNA Extraction, Library Construction and Illumina Sequencing

Total RNA was extracted from samples stored in the RNAstore using TRIzol reagent (Invitrogen Corporation, Carlsbad, CA, USA) following the manufacturer’s protocol. Total RNA was characterized and quantified using a Nano Drop and Agilent 2100 bioanalyzer (Thermo Fisher Scientific, MA, USA). Oligo (dT)-adhered magnetic beads were used to purify mRNA, and then fragmented with fragment buffer. cDNA was synthesized using the fragmented mRNA as a template, and the A-Tailing mix was added after repairing the end and connected with the RNA Index Adapters to construct the final library. Then the library was sequenced on the BGIseq500 platform (BGI-Shenzhen, China) to obtain 100 base paired-end reads.

### GO and KEGG Enrichment Analysis

All DEGs were annotated with GO and KEGG analysis. Gene ontology (GO) enrichment analysis of differentially expressed genes was implemented by the GOseq R package, in which gene length bias was corrected. KEGG is a database for understanding systematic gene function analysis and genomic information. The significant levels of terms and pathways were selected with a *P*-value of less than 0.05.

### Real-Time Quantitative PCR

Isolation of total RNA from cells at different time points using Trizol reagent. All RNA samples’ purity was detected by analyzing the A260/A280 ration using a Nano drop ND-1000spectrophotometer (Nano drop Technologies), which was expected to be 1.8~2.0. Total RNAs were reverse transcribed into cDNA with the ReverTra Ace qPCR RT Kit (Toyobo, Osaka, Japan); RT-qPCR was performed primarily according to a previous method (21). Target genes were detected using the previously described primers. All reactions were performed in triplicate and in at least three independent experiments. The relative levels of gene expression were determined with the 2−ΔCt method. All primer sequences are listed in **Table 1**.

**Table 1:** Primers for RT-qPCR analysis of gene expression.

Target Gene	Forward primer sequence	Reverse primer sequence	Accession no.
**IL-6**	TTCGACGAGGAGAAATGCTT	CCTTATCGTCGTTGCCAGAT	XM_013100522.2
**IL-1β**	AAAACGCTCTTCGTGCTGTC	CTCCTGCTGCTCTTCCTCAC	DQ393268
**IFN-β**	TCTACAGAGCCTTGCCTGCAT	TGTCGGTGTCCAAAAGGATGT	KM035791.2
**MX**	TGCTGTCCTTCATGACTTCG	GCTTTGCTGAGCCGATTAAC	NM_001310409.1
**OASL**	AGTTTGACATTGCCCAGTCC	TCCTCCTCGTGATTCCATTT	KY775584
**CCL21**	GGAGAAGCAGAAGAACCCCC	GGGAAAGCATCCGTCCTCTC	DR764376
**β-actin**	GCCCTCTTCCAGCCATCTTT	CTTCTGCATCCTGTCAGCGA	EF667345.1

### Virus Infection and Determination of Fluorescence Formation Units and Tissue Culture Infectious Dose 50

The required dose of virus was diluted in the medium used to culture the various cell types for viral infection. The culture medium was removed after incubation for 1 h at 37°C in 5% CO2. The cells were washed twice with PBS and then maintained in the corresponding medium containing 1% FBS and 1% penicillin-streptomycin. The culture supernatant of virus-infected cells was collected and titrated to determine the tissue culture infectious dose 50 (TCID50) on DEFs for DPV, or FFU on BHK21 for VSV-GFP.

### Cytotoxicity Assay

DEF cells were treated with different concentrations of each inhibitor or the solvent DMSO for 24 h and the cell viability of DEF cells was determined with a Cell Titer 96 Non-Radioactive Cell Proliferation Assay kit (MTT, Promega Corp., Madison, WI).

### Antiviral Activity Assay

Cells were first treated with different concentrations of the inhibitors SP600125 (10 μmol/mL), LY3214996 (5 μmol/mL), TAK-715 (5 μmol/mL), BAY-117082 (5 μmol/mL), and Ruxolitinib (10 μmol/mL) at 37°C for 1 h, the cells were washed three times with PBS. The same doses of viruses DPV (CHv strain), DPV (CHa strain) or VSV-GFP were incubated with cells at 37°C for 1h, washed three times with PBS, and then treated with different concentrations of inhibitors SP600125, LY3214996, TAK -715, BAY-117082. Cell supernatants were collected for TCID50 or FFU assay. GFP expression was detected under fluorescence microscopy to observe VSV-GFP and DPV virus plaque formation.

### Statistical Analysis

Data are expressed as the mean and standard error of the mean (SEM), and the significance of differences between groups was evaluated using the students’ t-test or one-way analysis of variance followed by Tukey’s *post-hoc* test. Results with *P < 0.05, **P < 0.01, ***P < 0.001, and ****P < 0.0001 were considered to be statistically significant.

## Results

### Significantly Differentially Expressed Genes After CHv and CHa Infection

The second-generation sequencing RNA-seq has been widely used in oncology, immunology, and cell biology in recent years (22, 23). To clarify the differences in gene expression in the duck immune cells, we applied the RNA-seq transcriptome sequencing technology to analyze the gene expression differences in the duck monocytes/macrophages cell model infected with DPV CHv strain or CHa strain at 48 hpi.

611 million read number or read counts were generated from nine samples. To ensure ideal results for subsequent genomic mapping and differential gene change analysis, raw reads were filtered to remove low-quality data, resulting in a total of 574 million (574 660 000) clean reads, achieving high-quality eukaryotic transcriptome reconstructions of the standard, an average of 53.13% of which mapped to the duck reference genome (**Table 2**). The raw sequencing data of the control group, CHv infected group and CHa infection group have been deposited into the Short Reads Archive (SRA) database under the accession numbers, SRR18825687, SRR18825689 and SRR18825688, respectively.

**Table 2:** The number of reads of all bases detected using RNA-seq in DPV-infected and control cells.

Library	Number of raw reads (M)	Total Clean reads (M)	Number of uniquely mapped reads (M)	Uniquely Mapping (%)
CHa-1	67.68	63.73	36.587393	57.41
CHa-2	67.68	63.73	36.561901	57.37
CHa-3	67.68	63.66	36.496278	57.33
CHv-1	67.68	63.28	24.691856	39.02
CHv-2	67.68	63.52	25.719248	40.49
CHv-3	67.68	63.31	25.621557	40.47
Mock-1	67.56	63.85	39.49761	61.86
Mock-2	70.19	65.91	40.409421	61.31
Mock-3	67.68	63.67	39.742814	62.42
Total	611.51	574.66	305.328078	

**Figure 1:**
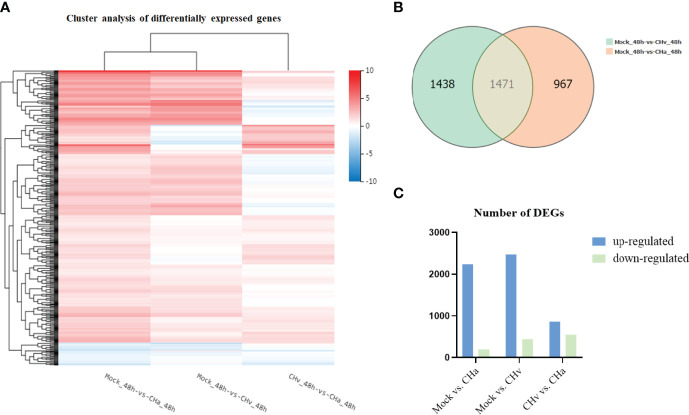
Transcriptome data profile generated by BGIseq-500 platform and differential expression analysis. **(A)** Heat map analysis is used to classify gene expression patterns at 48 hpi. Genes with similar expression patterns were clustered, as shown in the heat map. To intensity of the color indicates gene expression levels that were normalized according to log10 (FPKM + 1) values. Red represents high expression level genes and blue represents low expression level genes. **(B)** Venn diagram displaying a global view of the numbers of differentially expressed genes. The overlap of differentially expressed genes at 48hpi Mock vs. CHa and Mock vs. CHv. The numbers in the diagram indicate gene numbers and refer to each comparison. **(C)** Histogram showing screened differentially expressed genes.

The heat map visually compares the overall level of differential expression genes (DEGs), as shown in **Figure 1A**, the regulation of gene expression was at a completely different level in the CHv strain infection, CHa strain infection, or control groups. By Venn analysis the DEGs were further divided into three main parts (**Figure 1B**). There are 1438 DE genes in the green part representing the number of genes expressed only in CHv infected cells, while the red part has 967 DEGs that are expressed only in CHa infected cells. Compared with the control group, the intersection part with 1471 DE genes was co-expressed in CHv and CHa infected groups.

To comprehensively understand the changes in gene expression between different treatment groups, we performed three pairwise comparisons (Mock vs. CHa, Mock vs. CHv, CHv vs. CHa). DEGs were identified by p-value < 0.05 (**Figure 1C**). There were 2,438 DEGs significantly differentially expressed in Mock vs. CHa, which include 2,239 up-regulated genes and 199 down-regulated genes. Among 2,909 DEGs in Mock vs. CHv group, the up-regulated genes were 2,468 and the down-regulated genes were 441. Moreover, there were 1,399 DEGs in CHv vs. CHa group, among which 858 were up-regulated genes and 541 were downregulated genes. At 48 hpi, DEGs were associated with innate immune and inflammatory responses and may play an important role in the host’s defense response to DPV infection. Furthermore, these results cluster the samples by differential processing and are visible by constructing volcano plots of DEGs.

### Analysis of GO Annotation and KEGG Pathway

**Figure 2:**
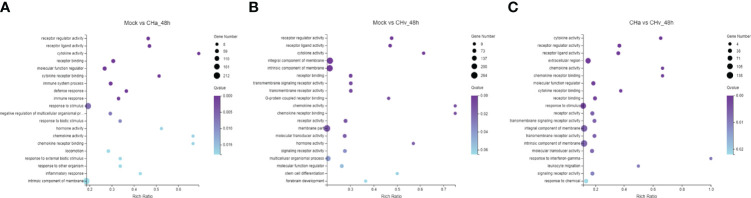
Top 20 Gene ontology (GO) terms of DEGs expressed in DPV CHv or CHa infected MM cells. The top 20 GO terms were selected according to *p* -value < 0.05. **(A)** Mock and CHa. **(B)** Mock and CHv. **(C)** CHa and CHv.

To confirm which biological processes were involved in DPV infection and those that were unidentified previously, the collected DEGs were categorized into three functional groups according to Gene Ontology (GO): Cellular Composition (CC), Molecular Function (MF) and Biological Process (BP). The GO analysis and enrichment results of the transcripts from the 3 groups were similar (**Supplementary Figures S1A–C**). To select useful genes for further exploration, 30 significant GO terms were listed (**Figures 2A– C**). Functional analysis showed that the top three important GO terms for DEGs expressed in CHa strain infected at 48hpi were intrinsic component of membrane, stimulus responsiveness, and molecular function regulator; The top three important GO terms of DEGs expressed in CHv infected were membrane part, an integral component of the membrane and intrinsic component of membrane. The top three important GO terms for DEGs expressed in both the CHa infected group and the CHv infected group were stimulus responsiveness, integral membrane component, and intrinsic membrane component.

The KEGG database is used for pathway-based classification of orthologous genes, providing useful information for predicting genes’ biological processes and phenotypic characteristics (24). The KEGG database was used to map DEGs to reference canonical signaling pathways in monocytes/macrophages after infection with DPV strains. The results showed that DEGs were mainly related to metabolism, viral infectious diseases, endocrine system, immune system, transcription, replication repair, and signaling transduction (**Supplementary Figures S2A–C**).

**Figure 3:**
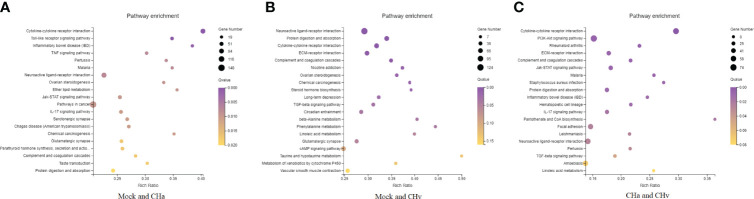
Top 20 KEGG pathways in DPV CHv or CHa infected MM cells. **(A)** KEGG pathways of Mock vs. CHa. **(B)** KEGG pathways of Mock vs. CHv. **(C)** KEGG pathways of CHa vs. CHv.

Next, to deeply investigate the functions of these DEGs, KEGG pathway/enrichment analysis was performed. As shown in **Figures 3A–C**, the top 20 enriched KEGG pathways are listed based on P<0.05. The DEGs identified in the uninfected and DPV CHa strain-infected groups were mainly involved in cytokine-cytokine receptor interaction, Toll-like receptor signaling pathway, inflammatory bowel disease, and TNF signaling pathway. The DEGs identified in the uninfected and DPV CHv-infected groups mainly involved interactions between neuroactive ligands and receptors, protein digestion and absorption, interactions between cytokines and cytokine receptors, and interactions between ECM and receptors. However, the DEGs identified in CHa and CHv infection groups mainly involved cytokine-cytokine receptor interaction, PI3K-AKT pathway, ECM-receptor interaction, and JAK-STAT signaling pathway. Here, we listed the signaling pathways associated with infection with DPV in the transcriptome of duck monocyte/macrophage cell models (**Table 3**). We found that DPV virus-infected cells significantly activated MAPK and NF-kappa B signaling pathways in host cells. This finding suggests that viruses and host cells utilize different strategies that may be involved in DPV infection-induced pathogenesis.

**Table 3:** Associated with signal transduction in the transcriptome of duck monocytes or macrophages infected with DPV at 48hpi.

Description	P-value	Corrected P-value	Number of DEG
**DPV CHv vs. MOCK**			
PI3K-Akt signaling pathway	0.05175328	0.4983102	104
cAMP signaling pathway	0.004924622	0.09762339	67
MAPK signaling pathway	0.3974499	0.979198	67
Rap1 signaling pathway	0.2252467	0.8162165	65
Jak-STAT signaling pathway	0.7118586	0.9999972	35
TGF-beta signaling pathway	0.001056776	0.03237577	33
TNF signaling pathway	0.3560591	0.9523168	22
Phosphatidylinositol signaling system	0.9378876	0.9999972	19
NF-kappa B signaling pathway	0.7776547	0.9999972	18
mTOR signaling pathway	0.9999857	0.9999972	16
**DPV CHa vs. MOCK**			
PI3K-Akt signaling pathway	0.00765817	0.0647999	98
MAPK signaling pathway	0.002785986	0.03405094	76
Rap1 signaling pathway	0.01756472	0.1114684	66
Jak-STAT signaling pathway	0.000293616	0.00950868	52
cAMP signaling pathway	0.08399132	0.3079682	52
TNF signaling pathway	0.00013179	0.007467114	33
TGF-beta signaling pathway	0.007921306	0.06535077	27
NF-kappa B signaling pathway	0.01656176	0.1114684	27
mTOR signaling pathway	0.9960666	1	19
Phosphatidylinositol signaling system	0.9810913	1	14

### Viruses Induced the Expression of Cytokine in Host Cells

**Figure 4:**
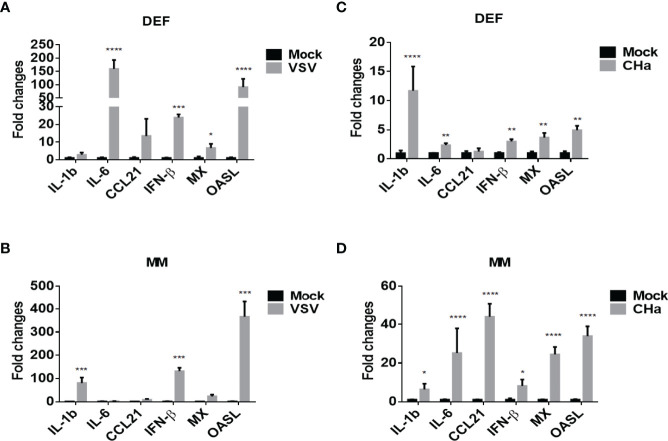
VSV and DPV CHa strain-induced IFN, ISGs and cytokines in duck DEF and MM cells. DEF cells were infected with VSV **(A)** or DPV CHa strain **(C)** at 5 MOI for 24 h. MM cells were infected with VSV **(B)** or DPV CHa strain **(D)** at 5 MOI for 24 h. The expression level of IL-1β, IL-6, CCL21, IFN-β, MX and OASL were tested using RT-qPCR 24 h post-treatment. The relative expression was presented as fold changes compared to mock treatment. “*” was considered significant difference (*p* < 0.05); “**” was considered highly significant difference (*p* < 0.01); “***” was considered highly significant difference (*p* < 0.001); “****” was considered highly significant difference (*p* < 0.0001).

It was reported that cytokines are key modulators of inflammation, participating in acute and chronic inflammation *via* a complex and sometimes seemingly contradictory network of interactions (25). To determine whether DPV infection induces cytokine expression in host cells, we infected duck monocyte/macrophage cells with CHa virulent strain at 5 MOI and examined the mRNA expression of IL-1β, IL-6, CCL21, IFN-β, MX, and OASL at 24 hpi. We found the expression of inflammatory cytokines IL-1β, IL-6, CCL21, type I interferon β (IFN-β), and interferon-stimulated genes (ISGs) MX and OASL were upregulated in CHa strain infected monocyte/macrophage cells (**Figure 4D**). Vesicular stomatitis virus (VSV) is a prototypical enveloped animal virus that has been widely explored as a virus model to assess the antiviral activity of IFN due to its broad host range and robust replication properties in a variety of mammalian and insect cells (26). Here, we found that the expression of IL-1β, IFN-β and OASL was significantly induced in VSV infected monocytes/macrophage cells (**Figure 4B**). In addition, similar results were obtained when duck embryo fibroblasts (DEFs) were infected with CHa strain or VSV at the 5 MOI (**Figures 4A, C**). These results demonstrated that CHa induces the production of inflammatory cytokines, type I interferons, and ISGs in duck cells.

### Inflammatory Pathways Are Involved in Viral Induction of Cytokines

Pattern recognition receptors (PRRs) that sense pathogen-associated molecular patterns, are responsible for recognizing invading viruses, and then initiating antiviral innate immune responses. Following the engagement of these PRRs, type I and III interferons (IFN), chemokines and proinflammatory cytokines are produced to activate inflammation (27). The above results indicate that DPV virus-infected cells significantly activate the MAPK and NF-kappa B signaling pathways in host cells.

**Figure 5:**
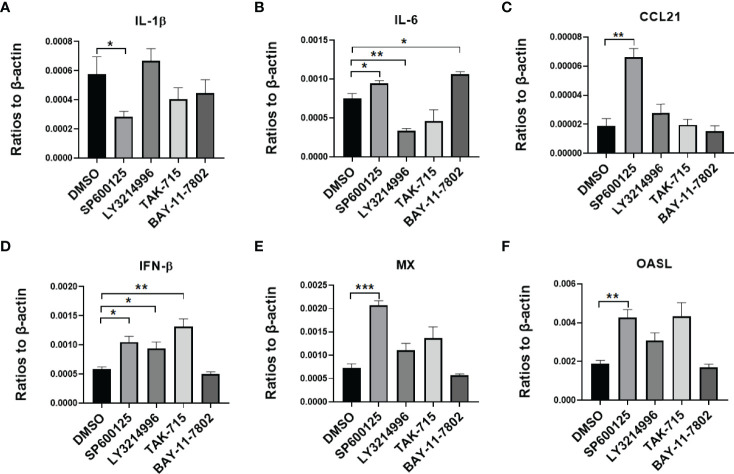
IFN, ISGs, and cytokines were regulated by inflammatory signaling in duck DEF cells infected with the CHa strain. DEF cells were pretreated with SP600125 (JNK inhibitor), LY3214996 (ERK inhibitor), TAK-715 (p38 inhibitor) and BAY-117082 (NF-kB inhibitor) for 1 h at 10, 5, 5 and 5 M respectively, DMSO as control, then the cells were infected with CHa strain at 5 MOI for 1 h, then the same concentration of inhibitor was added. The expression level of IL-1β **(A)**, IL-6 **(B)**, CCL21 **(C)**, IFN-β **(D)**, MX **(E)**, and OASL **(F)** were tested using RT-qPCR 24 h post-treatment. The relative expression was presented as ratios to β-actin compared to mock treatment. “*” was considered significant difference (*p* < 0.05); “**” was considered highly significant difference (*p* < 0.01); “***” was considered highly significant difference (*p* < 0.001).

**Figure 6:**
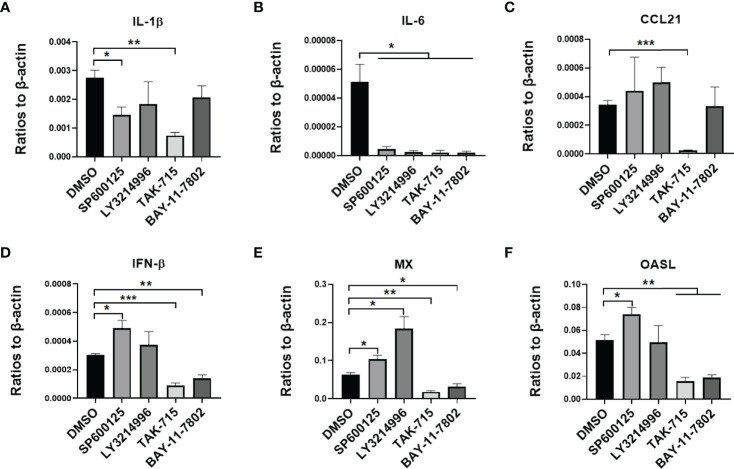
IFN, ISGs, and cytokines were regulated by inflammatory signaling in duck MM cells infected with the CHa strain. MM were pretreated with SP600125 (JNK inhibitor), LY3214996 (ERK inhibitor), TAK-715 (p38 inhibitor) and BAY-117082 (NF-kB inhibitor) for 1 h at 10, 5, 5 and 5 M respectively, DMSO as control, then the cells were infected with CHa strain at 5 MOI for 1 h, then the same concentration of inhibitor was added. The expression level of IL-1β **(A)**, IL-6 **(B)**, CCL21 **(C)**, IFN-β **(D)**, MX **(E)**, and OASL **(F)** were tested using RT-qPCR 24 h post-treatment. The relative expression was presented as ratios to β-actin compared to mock treatment. “*” was considered significant difference (*p* < 0.05); “**” was considered highly significant difference (*p* < 0.01); “***” was considered highly significant difference (*p* < 0.001).

To testify that MAPK and NF-kB pathways impact virus infection of duck cells, we compared cytokine transcriptional levels after blocking of JNK, ERK, p38, and NF-kB pathways with specific inhibitors. Firstly, the duck monocytes/macrophages were pretreated with SP600125 (JNK inhibitor,10 μmol/mL), LY3214996 (ERK inhibitor, 5 μmol/mL), TAK-715 (p38 inhibitor, 5 μmol/mL), BAY-11-7802 (NF-KB inhibitor, 5 μmol/mL), or solvent DMSO for 1 h, and then infected with 5 MOI of the CHa strain for another 1 h, after infection the cells were washed with PBS and the same concentration of each inhibitor was added. Cell samples were harvested at 24 hpi, and the transcript levels of cytokines were detected by RT-qPCR. We observed that SP600125 promoted the expression levels of IL-6, CCL21, IFN-β, MX, and OASL, but the expression level of IL-1β decreased in DEF cells in the presence of CHa strain infection (**Figures 5A–F**). In monocytes or macrophages, SP600125 treatment also promoted the expression levels of IFN-β, MX, and OASL, but not CCL21, under CHa strain infection (**Figures 6A–F**). However, the expression levels of IL-1β and IL-6 were decreased by SP600125 treatment in monocytes or macrophages in the presence of CHa strain infection.

Next, the same experiment was performed with the VSV-GFP model virus at the same MOI. We found that four pathway inhibitors reduced the expression level of IL-1β, but not IL-6 or CCL21, in duck monocytes/macrophages (**Supplementary Figures S3A–C**). Besides, we observed that the expression levels of IFN-β, MX, and OASL expression levels were promoted by SP600125 treatment in duck monocytes/macrophages and VSV infection (**Supplementary Figures S3D–F**). Similar results were observed in DEF cells (**Supplementary Figures S4A–F**). Thus, we proposed that SP600125 may promote IFNR signaling in cells in the context of viral infection. Altogether, these data demonstrated that the JNK, ERK, p38 and NF-kB pathways all regulate intracellular cytokines in the case of virus infection in duck cells. Interestingly, virus-induced cytokine expression was significantly enhanced in different cell types treated with SP600125. These results suggest that SP600125 strengthens the anti-viral responses of host cells.

### SP600125 Inhibits DPV CHv Infection in Different Duck Cell Types

**Figure 7:**
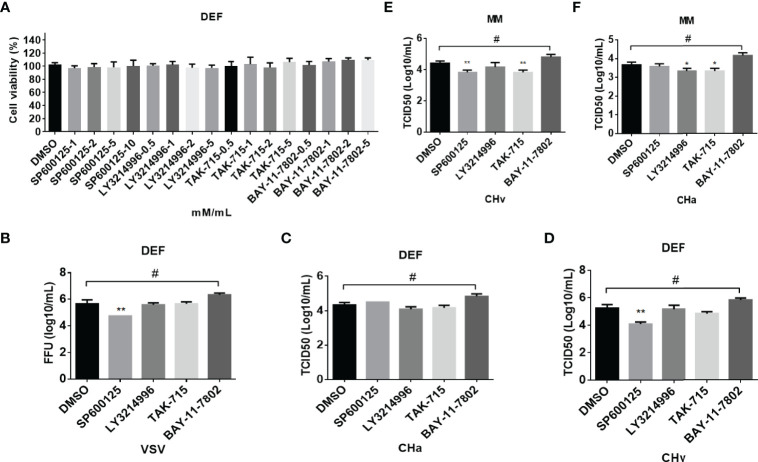
The inflammatory signaling affects virus replication in DEF and MM cells. **(A)** The cell toxicity of the JNK, ERK, p38, and NF-kB inhibitor was determined through MTT. **(B–F)** The viral titer was detected. DEF cells were pretreated with SP600125 (JNK inhibitor), LY3214996 (ERK inhibitor), TAK-715 (p38 inhibitor) and BAY-117082 (NF-kB inhibitor) for 1 h at 10, 5, 5, and 5 M respectively, DMSO as control, then the cells were infected with VSV **(B)**, CHv **(C, E)** and CHa strain **(D, F)** at 1 MOI for 1 h, then the same concentration of inhibitor was added. At 24 hpi, the cell culture supernatants were collected and the FFU of VSV-GFP and the TCID50 of CHa or CHv were determined on BHK21 or DEF cells. “*” was considered significant difference (*p* < 0.05); “**” was considered highly significant difference (*p* < 0.01). "*" indicates significantly down-regulated group compared to DMSO group, "#" indicates up-regulated group compared to DMSO group.

Cytokines play an important role in the antiviral response of host cells. Firstly, the cell viability was detected with an MTT assay after the cells were treated with different doses of SP600125, LY3214996, TAK-715 or BAY117082.That data showed no obvious cell death induced by these four inhibitors (**Figure 7A**). Therefore, the antiviral ability of SP600125 is independent of cell death.

The above results showed that SP600125 treatment significantly promotes cytokine expression, which implied that SP600125 treatment may inhibit the growth of the virus in different types of cells. To clarify this hypothesis, cells were infected with DPV at 1 MOI, with or without the addition of SP600125, LY3214996, TAK-715, BAY-11-7802, or DMSO. The viral load in the cell culture supernatants was measured at 24 hpi. As shown in **Figures 7B–D**, the viral load in the supernatant was significantly reduced after SP600125 treatment compared to the DMSO controls, indicating that SP600125 treatment restricted the growth of VSV-GFP and CHv strain in DEF cells but did not affect the growth of CHa strain. Subsequently, we observed that the viral load in the supernatant was significantly reduced after SP600125 treatment compared to the DMSO controls, indicating that SP600125 restricted the growth of CHv, but not CHa in monocytes/macrophages (**Figures 7E–F**). Altogether, these data suggest that the JNK pathway plays a critical role in VSV and DPV infection in duck cells.

### SP600125 Enhances IFN and IFN-Stimulated Gene (ISG) Expression, but Inhibits Inflammatory Responses in DPV-Infected DEF Cells

**Figure 8:**
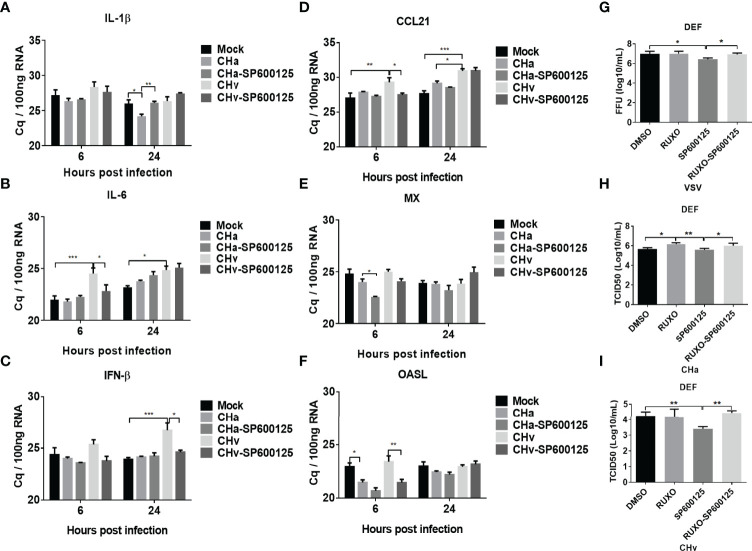
JNK signaling regulates IFN signaling and virus replication. The inflammatory signaling affects virus replication in DEF cells. **(A-F)** DEF cells were pretreated with SP600125 (JNK inhibitor) for 1 h at 10 μM, DMSO as control, then the cells were infected with CHa or CHv strain at 5 MOI for 1 h, then the same concentration of inhibitor was added. The expression level of IL-1β, IL-6, CCL21, IFN-β, MX, and OASL were tested using RT-qPCR at 6 and 24 h post-treatment. The relative expression was presented as Cq values compared to mock treatment. **(G-I)** The DEF cells were pretreated with SP600125 (JNK inhibitor) and (or) Ruxolitinib (IFNR inhibitor) for 1 h at 10 μM, then the cells were infected with VSV, CHa or CHv strain at 1 MOI for 1 h, then the same concentration of inhibitor was added. The cell culture supernatant was collected and the viral titer was determined on DEF cells. “*” was considered significant difference (*p* < 0.05); “**” was considered highly significant difference (*p* < 0.01); “***” was considered highly significant difference (*p* < 0.001).

We focused on the antiviral mechanism of SP600125 in duck cells. We further examined the modulation of IFN signaling under CHa strain or CHv strain infection and combined with SP600125 treatment. Since SP600125 inhibited DPV infection, we infected DEF cells with 5 MOI of CHa strain or CHv strain for 1 h, and SP600125 was added to treat cells for 6 or 24 h. The transcription levels of cytokines IL-1β, IL-6, CCL21, IFN-β, MX and OASL were detected at 6h and 24h. Since DPV infection causes obvious cellular lesions and changes the expression level of the housekeeping genes, so we chose the Cq value to make the map. As shown in **Figures 8A– F**, the IFN-β level was reduced in CHv strain infection cells. However, the SP600125 treatment reversed this phenomenon of IFN-β at the late time point of CHv infection, indicating that inhibiting the activation of the JNK pathway will be beneficial to the regulation of the IFN signaling pathway under virus infection.

Next, we explored the impact of the JNK pathway on viral replication. Here we applied the IFNR-specific inhibitor Ruxolitinib to block IFN signaling in DEF cells to examine the specificity of the effect of JNK pathway inhibitors on viral replication. Under these conditions, we found that VSV and CHv strain replication in DEF cells was inhibited in SP600125-treated cells, but virus titers were recovered in SP600125 and Ruxolitinib combination treatment cells (**Figures 8G, I**). We also found that Ruxolitinib alone promoted the replication of the CHa virus, while SP600125 alone did not affect on the replication of CHa vaccination (**Figure 8H**). In conclusion, our results suggest that SP600125 acts as an antiviral inhibitor in host cells against DPV CHv virulent strain by inhibiting the JNK MAPK signaling pathway to enhance the expression of type I interferon.

## Discussion

Duck plague, caused by duck plague virus (DPV), is an acute hemorrhagic disease that causes huge economic losses to the poultry industry worldwide due to reduced egg production and high mortality in infected ducks (28). Therefore, deepening the understanding of the molecular mechanisms of the host-pathogen interaction is of great significance in suppressing the occurrence and prevalence of DPV infection. In recent years, RNA-Seq technology has become a powerful tool that provides high-resolution expression and high-sensitivity measurements when detecting low-abundance transcripts, and has also been used to determine the mechanism of action of DPV (29–31). However, there is no assay executed in duck immune cells which is unavailable before. The previous study successfully established the duck monocyte/macrophages models for duck pathogenesis research (20). RNA-Seq was used to study molecular expression profiles in the monocytes/macrophages and further to gain insight into the immune response patterns of DPV.

Raw reads were filtered to remove low-quality data, coming with 574 million clean reads. DEGs were classified and annotated by GO and KEGG signaling pathway analysis. By comparing the transcriptome data of the libraries, the results showed that most of the DEGs were associated with cytokine-cytokine receptors, the immune system and signal transduction. At 48hpi, the regulation of the MAPK signaling pathway (CHv: 67 genes, CHa: 76 genes) and the NF-κB signaling pathway (CHv: 18 genes, CHa: 27 genes) were affected, implying an alteration of the inflammatory response (**Table 3**).

Excessive inflammation is becoming a critical factor in many viral infectious diseases. MAPKs are important regulators for inflammatory cytokine and chemokine expression (32, 33). Cytokines are a class of small molecular weight soluble glycoproteins or protein polypeptides that are stimulated and secreted by immune cells and some non-immune cells, and play critical roles in many cellular biological processes (34). From the results of the KEGG analysis in this study, we found that the expression of many important cytokines and cytokine receptor genes, CHv-infected and CHa-infected groups. Viral infection usually induces the expression of interferons, interferon-stimulating factors, chemokines and cytokines. Excessive expression of such proteins may have irreversible effects on the host (35). For instance, this “cytokine storm” can lead to severe pathological damage and high mortality (36, 37). Our results showed that the transcript levels of IL-1β, IL-6, CCL21, IFN-β, MX and OASL in DPV CHa strain-infected monocytes/macrophages were 2-40-fold higher than those in the mock-infected group, while the transcript levels of IFN-β and OASL in VSV virus-infected monocytes/macrophages were 80-300-fold higher than those in the mock-infected group. In addition, similar results were obtained when duck embryo fibroblasts were infected with CHa strain or VSV virus at the same MOI. These results suggested that cytokines play an important role in host responses against the infection of CHa strain or VSV.

Viruses usually modulate the immune system of host cells in the early or middle stage of infection, when most of the signaling pathways are activated by the cells to fight the invading pathogen (38). Therefore, we mainly analyzed the influence of DPV invasion on the signaling pathway in host cells at 48hpi, and we found that the signaling pathways including JAK-STAT, MAPK, and NF-κB, were involved in CHv strain and CHa strain infection at 48hpi (**Figure 5**). The JAK/STAT signaling pathway is a ubiquitously expressed intracellular signal transduction pathway involved in crucial biological processes, including cell proliferation, differentiation, apoptosis, and immune regulation (39). PRRSV nonstructural protein 1β induces degradation of nuclear membrane protein a1, thereby blocking nuclear translocation of STAT1 and STAT2, leading to the inhibition of the interferon-activated JAK-STAT signaling (40). In monocytes/macrophages, JNK, P38 and ERK1/2 of MAPKs are involved in TLR-induced IL-10 production and are tightly controlled by IFN-γ (41). The nuclear factor NF-κB pathway has long been regarded as a typical pro-inflammatory signaling pathway, which participates in the immune regulation and inflammatory response and participates in cell cycle regulation, cell differentiation, and apoptosis (42). Human papilloma virus (HPV) down-regulated NF-κB to eliminate the inhibitory activity of the immune system on its replication, ultimately leading to a persistent HPV infection state (43).

Some viruses exploited MAPK signaling to sustain excessive inflammation for their replication. Our transcriptome sequencing results found that virus infection leads to activation of the MAPK signaling pathway, but the addition of JNK pathway inhibitor SP600125 leads to up-regulation of innate immunity. In addition, similar results were obtained in the DEF cells and monocytes/macrophages infected with VSV virus. These results suggest that the JNK pathway regulates expression of cytokines under viral infection conditions. Herpes simplex virus (HSV), Epstein-Barr virus (EBV), and varicella-zoster virus (VZV) activate the JNK pathway through various mechanisms, resulting in different consequences on infected cells. For example, in neurons, sustained activation of JNK by VZV favors viral lytic infection (44), HSV-2 infection increases JNK phosphorylation, and JNK signaling is involved in HSV-2-induced TLR9 transcriptional activation (45). EBV activates NF-κB and JNK *via* LMP1 (latent membrane protein 1), which is essential for the oncogenic activity of LMP1 (46). JNK/AP-1 signaling pathway is involved in PRRSV N protein-induced SOCS1 production, thereby promoting PRRSV replication. We further tested the antiviral activity of JNK pathway inhibitor SP600125 and found that it inhibited DPV CHv infection in MM cells, and VSV and DPV CHv infection in DEF cells. We postulated that SP600125 has broad-spectrum antiviral activity and can inhibit RNA and DNA virus infection in different duck cell types.

Our study shed broad spectra light on the transcriptome changes of monocytes/macrophages response to DPV infection. The transcriptome analysis results showed that DEGs related to signal transduction, including the MAPK signaling pathway, NF-κB signaling pathway and JAK-STAT signaling pathway, were involved in DPV infection. In addition, we found that activating the JNK signaling pathway inhibits the expression of cytokines in host cells and promotes viral replication, suggesting that DPV may take over the JNK signaling pathway for its benefit and that MAPK JNK may be a new target for antiviral drugs design. Altogether, our data provide new insights into signaling pathways and the potential responses of cytokines to DPV infection, broaden the activation of signaling pathways by a viral infection and help us better understand the molecular mechanisms underlying pathogen-host interactions.

## Data Availability Statement

The datasets presented in this study can be found in online repositories. The names of the repository/repositories and accession number(s) can be found below: Short Reads Archive (SRA) database under the accession numbers: SRR18825687, SRR18825689 and SRR18825688.

## Ethics Statement

The animal study was reviewed and approved by the Experimental Procedures and Animal Welfare Committee of Sichuan Agricultural University (approval permit number SYXK 2019-187).

## Author Contributions

LP and BT carried out the experiment. AC and MW conceived of and supervised the study. AC, RJ, DZ, QY, YW, JH, XZ, SC and SZ provided ideas contributing to the conception of this article. XO, SM, QG and DS helped to draw the pictures. MW modified the article. All authors contributed to the article and approved the submitted version.

## Funding

This work was supported by China Agriculture Research System of MOF and MARA, and the Sichuan Veterinary Medicine and Drug Innovation Group of China Agricultural Research System (SCCXTD-2020-18).

## Conflict of Interest

The authors declare that the research was conducted in the absence of any commercial or financial relationships that could be construed as a potential conflict of interest.

## Publisher’s Note

All claims expressed in this article are solely those of the authors and do not necessarily represent those of their affiliated organizations, or those of the publisher, the editors and the reviewers. Any product that may be evaluated in this article, or claim that may be made by its manufacturer, is not guaranteed or endorsed by the publisher.

## Supplementary Material

Supplementary Figure 1 Gene ontology (GO) terms of DEGs expressed in DPV CHv or CHa infected MM cells. GO terms were classified into 3 categories, including cellular component (CC), molecular function (MF), and biological process (BP). **(A)** GO annotation of DEGs expressed in Mock and CHa. **(B)** GO annotation of DEGs expressed in Mock and CHv. **(C)** GO annotation of DEGs expressed in CHa and CHv.

Supplementary Figure 2 KEGG analysis of genes identified in each group at 48 hpi. **(A)** KEGG analysis of Mock vs. CHa. **(B)** KEGG analysis of Mock vs. CHv. **(C)** KEGG analysis of CHa vs. CHv.

Supplementary Figure 3 IFN, ISGs, and cytokines were regulated by inflammatory signaling in duck MM cells infected with the VSV-GFP model virus. MM cells were pretreated with SP600125 (JNK inhibitor), LY3214996 (ERK inhibitor), TAK-715 (p38 inhibitor) and BAY-117082 (NF-kB inhibitor) for 1 h at 10, 5, 5 and 5 μM respectively, DMSO was used as control, then the cells were infected with VSV at 5 MOI for 1 h, then the same concentration of inhibitor was added. The expression level of IL-1β, IL-6, CCL21, IFN-β, MX and OASL were tested using RT-qPCR 24 h post-treatment. The relative expression was presented as ratios to β-actin compared to mock treatment. “*” was considered significant difference (*p* < 0.05); “***” was considered highly significant difference (*p* < 0.001).

Supplementary Figure 4 IFN, ISGs, and cytokines were regulated by inflammatory signaling in duck DEF cells infected with the VSV-GFP model virus. DEF cells were pretreated with SP600125 (JNK inhibitor), LY3214996 (ERK inhibitor), TAK-715 (p38 inhibitor) and BAY-117082 (NF-kB inhibitor) for 1 h at 10, 5, 5 and 5 μM respectively, DMSO was used as control, then the cells were infected with VSV at 5 MOI for 1 h, then the same concentration of inhibitor was added. The expression level of IL-1β, IL-6, CCL21, IFN-β, MX and OASL were tested using RT-qPCR 24 h post-treatment. The relative expression was presented as ratios to β-actin compared to mock treatment. “*” was considered significant difference (*p* < 0.05); “**” was considered highly significant difference (*p* < 0.01); “***” was considered highly significant difference (*p* < 0.001).
