# Supplementary figures and images for: Duck Plague Virus Negatively Regulates IFN Signaling to Promote Virus Proliferation via JNK Signaling Pathway
Source: Front Immunol. 2022 Jun 28;13:935454. doi: 10.3389/fimmu.2022.935454 (PMC9275408; doi:10.3389/fimmu.2022.935454)

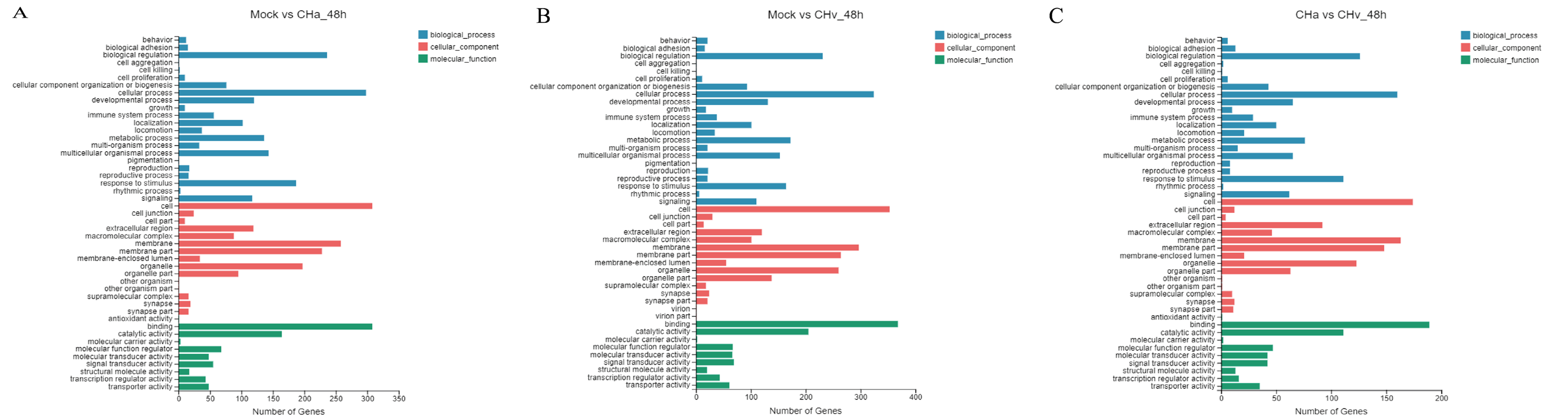

Supplement: Supplementary Figure 1 — Gene ontology (GO) terms of DEGs expressed in DPV CHv or CHa infected MM cells. GO terms were classified into 3 categories, including cellular component (CC), molecular function (MF), and biological process (BP). (A) GO annotation of DEGs expressed in Mock and CHa. (B) GO annotation of DEGs expressed in Mock and CHv. (C) GO annotation of DEGs expressed in CHa and CHv. [file Image_1.tif]

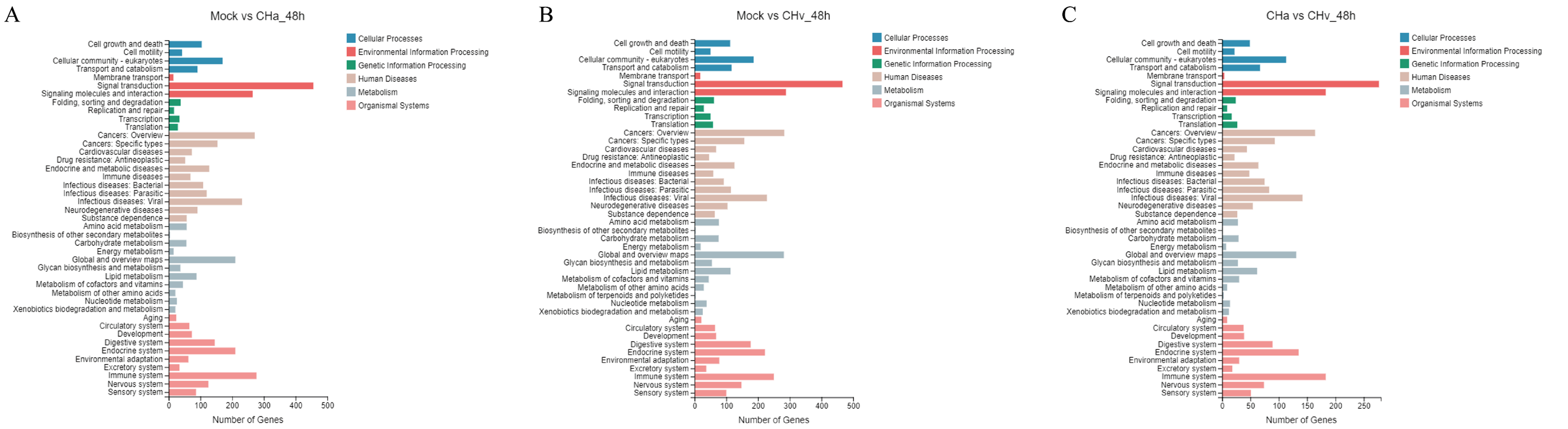

Supplement: Supplementary Figure 2 — KEGG analysis of genes identified in each group at 48 hpi. (A) KEGG analysis of Mock vs. CHa. (B) KEGG analysis of Mock vs. CHv. (C) KEGG analysis of CHa vs. CHv. [file Image_2.tif]

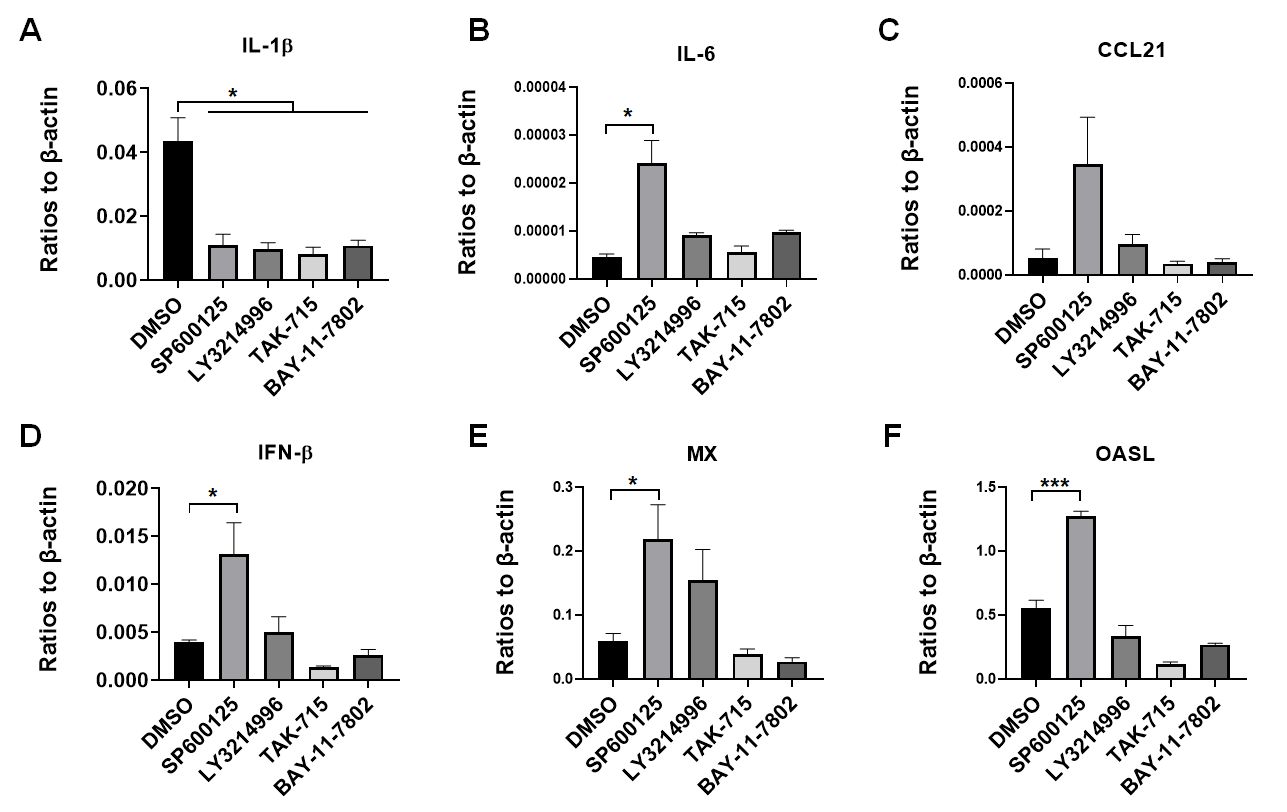

Supplement: Supplementary Figure 3 — IFN, ISGs, and cytokines were regulated by inflammatory signaling in duck MM cells infected with the VSV-GFP model virus. MM cells were pretreated with SP600125 (JNK inhibitor), LY3214996 (ERK inhibitor), TAK-715 (p38 inhibitor) and BAY-117082 (NF-kB inhibitor) for 1 h at 10, 5, 5 and 5 μM respectively, DMSO was used as control, then the cells were infected with VSV at 5 MOI for 1 h, then the same concentration of inhibitor was added. The expression level of IL-1β, IL-6, CCL21, IFN-β, MX and OASL were tested using RT-qPCR 24 h post-treatment. The relative expression was presented as ratios to β-actin compared to mock treatment. “*” was considered significant difference (p < 0.05); “***” was considered highly significant difference (p < 0.001). [file Image_3.tif]

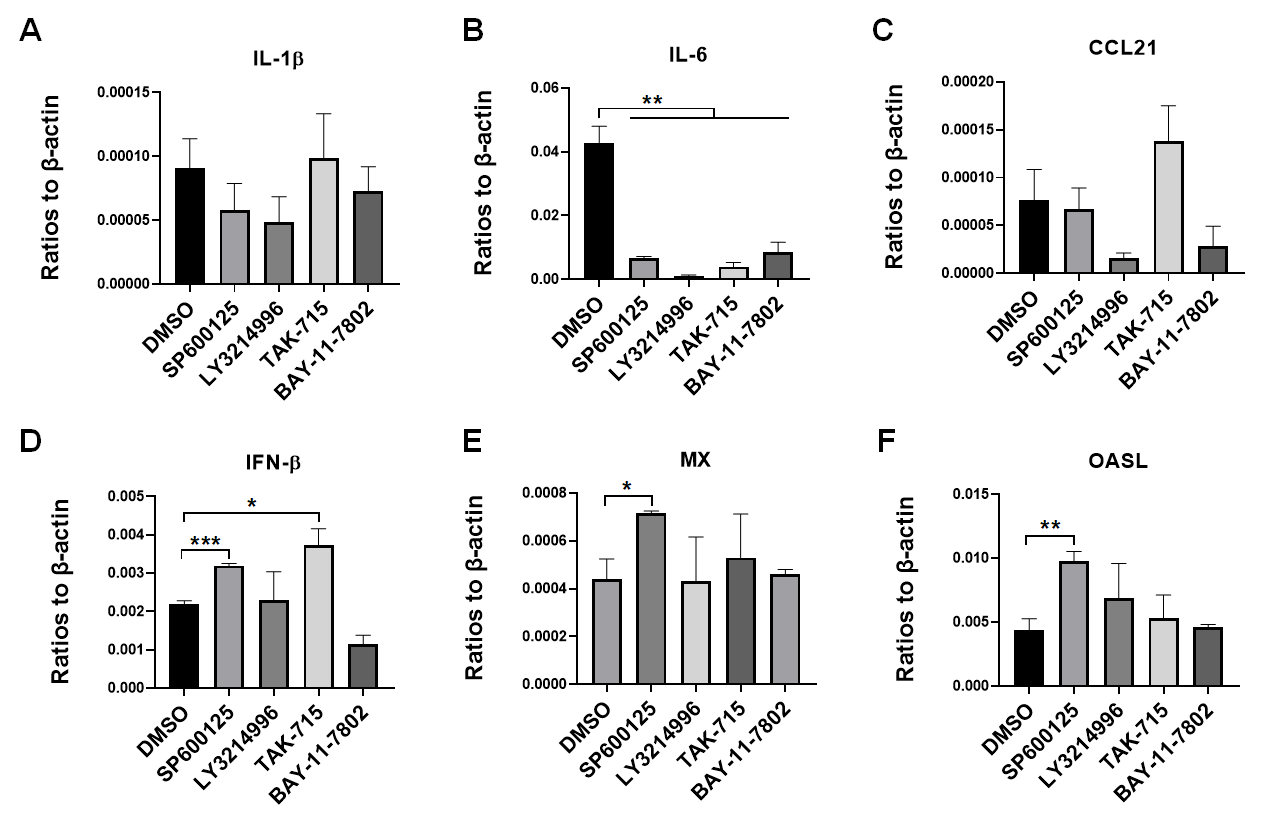

Supplement: Supplementary Figure 4 — IFN, ISGs, and cytokines were regulated by inflammatory signaling in duck DEF cells infected with the VSV-GFP model virus. DEF cells were pretreated with SP600125 (JNK inhibitor), LY3214996 (ERK inhibitor), TAK-715 (p38 inhibitor) and BAY-117082 (NF-kB inhibitor) for 1 h at 10, 5, 5 and 5 μM respectively, DMSO was used as control, then the cells were infected with VSV at 5 MOI for 1 h, then the same concentration of inhibitor was added. The expression level of IL-1β, IL-6, CCL21, IFN-β, MX and OASL were tested using RT-qPCR 24 h post-treatment. The relative expression was presented as ratios to β-actin compared to mock treatment. “*” was considered significant difference (p < 0.05); “**” was considered highly significant difference (p < 0.01); “***” was considered highly significant difference (p < 0.001). [file Image_4.tif]
